# Low molecular weight protein tyrosine phosphatase (LMWPTP) upregulation mediates malignant potential in colorectal cancer

**DOI:** 10.18632/oncotarget.3224

**Published:** 2015-03-14

**Authors:** Elmer Hoekstra, Liudmila L. Kodach, Asha M. Das, Roberta R. Ruela-de-Sousa, Carmen V. Ferreira, James C. Hardwick, C. Janneke van der Woude, Maikel P. Peppelenbosch, Timo L.M. ten Hagen, Gwenny M. Fuhler

**Affiliations:** ^1^ Department of Gastroenterology and Hepatology, Erasmus MC, University Medical Center Rotterdam, 's Gravendijkwal 230, NL-3015 CE Rotterdam, The Netherlands; ^2^ Department of Gastroenterology and Hepatology, Cancer Genomics Centre Netherlands and Centre for Biomedical Genetics, Leiden University Medical Center, Leiden, The Netherlands; ^3^ Department of Surgery, Section Surgical Oncology, Laboratory Experimental Surgical Oncology, Erasmus MC, University Medical Center Rotterdam, 's Gravendijkwal 230, NL-3015 CE Rotterdam, The Netherlands; ^4^ Department of Biochemistry, Institute of Biology, University of Campinas, Brazil (UNICAMP), Campinas, Sao Paulo, Brazil

**Keywords:** Colorectal cancer, kinases and phosphatases, signal transduction, metastasis

## Abstract

Phosphatases have long been regarded as tumor suppressors, however there is emerging evidence for a tumor initiating role for some phosphatases in several forms of cancer. Low Molecular Weight Protein Tyrosine Phosphatase (LMWPTP; acid phosphatase 1 [ACP1]) is an 18 kDa enzyme that influences the phosphorylation of signaling pathway mediators involved in cancer and is thus postulated to be a tumor-promoting enzyme, but neither unequivocal clinical evidence nor convincing mechanistic actions for a role of LMWPTP have been identified. In the present study, we show that LMWPTP expression is not only significantly increased in colorectal cancer (CRC), but also follows a step-wise increase in different levels of dysplasia. Chemical inhibition of LMWPTP significantly reduces CRC growth. Furthermore, downregulation of LMWPTP in CRC leads to a reduced migration ability in both 2D- and 3D-migration assays, and sensitizes tumor cells to the chemotherapeutic agent 5-FU. In conclusion, this study shows that LMWPTP is not only overexpressed in colorectal cancer, but it is correlated with the malignant potential of this cancer, suggesting that this phosphatase may act as a predictive biomaker of CRC stage and represents a rational novel target in the treatment of this disease.

## INTRODUCTION

Colorectal cancer (CRC) is one of the most common forms of cancer in the Western world. Although CRC mortality has been progressively declining since 1990, it still remains the second most common cause of cancer death in the US and Europe [1]. When in a non-metastatic state, surgery of the primary tumor is considered a curative treatment. Unfortunately, around 20% of the CRC patients already present with metastatic disease, dropping the 5-year survival rate from 90% to a dramatic 12% [2]. For this reason, treatments focusing on the prevention of this progression into the metastatic state are urgently called for.

All cellular functions are under tight control of the balance between phosphorylation and dephosphorylation of proteins. Like many neoplasms, disturbed protein phosphorylation patterns, indicating imbalanced kinase and/or phosphatase activities, are often observed in colorectal cancer [3]. So far, kinases have received most of the attention in cancer studies, as it is well established that deregulation of these enzymes can contribute to the development of multiple neoplasms [4]. Inhibitors of kinase activities, such as EGFR- and BRAF-inhibitors, are amongst the novel potential treatments currently explored for CRC. Despite the fact that these drugs have shown some promising results [5, 6], there is still a need for new, additional, classes of molecules as potential targets. Phosphatases could present such a class.

Generally assumed to be tumor suppressive by counteracting kinase activities, phosphatases have largely been ignored as viable targets for treatment. However, a tumor promoting role has also been suggested for certain phosphatases [7]. One of these is the ubiquitously expressed Low Molecular Weight Protein Tyrosine Phosphatase (LMWPTP), encoded by the gene *ACP1*. Enhanced mRNA expression of this phosphatase has been reported for some human tumors [8, 9]. This 18kDa protein tyrosine phosphatase can have a positive effect on cell growth and proliferation signaling by interacting with several molecules involved in these processes, such as Ephrin A2 receptor (EphA2) [10], β-catenin [11], platelet-derived growth factor receptor (PDGFR) [12], Janus kinase (JAK)-2 [13], and signal transducer and activator of transcription (STAT)-5 [14].

The aim of this study was to examine the expression levels and potential role of LMWPTP in colorectal cancer. Our study reveals a novel unexpected action of hypomethylation-mediated upregulation of LMWPTP mRNA and protein levels in primary colorectal cancer and shows that this upregulation mediates chemoresistance and increased migration that characterizes this infaust disease.

## RESULTS

### *ACP1* mRNA expression is increased in colorectal adenomas and carcinomas

**Figure 1:**
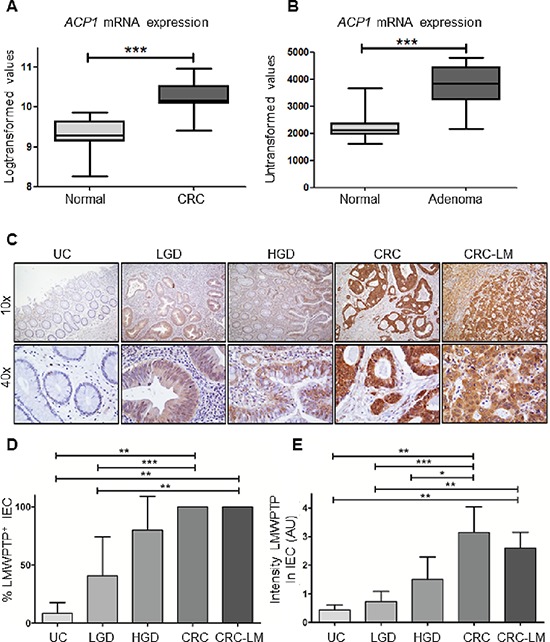
*ACP1* mRNA and LMWPTP protein expression are increased in colorectal dysplasia and carcinoma as compared to non-dysplastic tissue **(A, B)** Using publicly available gene expression data from Affymetrix platforms, *ACP1* expression (transcript 215227) was analyzed in carcinoma tissue (CRC) and adjacent normal colon tissue. Significantly higher expression of *ACP1* mRNA expression was observed in carcinoma tissues (*n* = 17, *P* = 0.0005 by student's *T*-test). Gene expression array comparing *ACP1* expression in colorectal adenoma to normal adjacent tissue shows increased *ACP1* mRNA expression in cancer tissues (*n* = 32, *P* < 0.0001) **(C)**. Tissues of patients with inactive ulcerative colitis (UC, *n* = 8), low grade dysplasia (*n* = 8), high grade dysplasia (HGD, *n* = 6), colorectal cancer (CRC, *n* = 12) and CRC liver metastasis (*n* = 5) were stained for LMWPTP by immunohistochemistry. Representative examples (10x and 40x magnifications) of UC, LGD, HGD, CRC and liver metastasis are shown. **(D, E)** LMWPTP staining was scored for percentage of positive intestinal epithelial cells as well as intensity of staining and statistical analysis was performed using Mann-Whitney *t*-test. (*P > 0.05; ** *P* > 0.01, *** *P* > 0.001).

To understand the role of LMWPTP in colorectal cancer, we first investigated the gene expression levels of *ACP1* using publicly available microarray datasets from Affymetrix Platforms. Expression of the LMWPTP encoding gene *ACP1* (transcript 215227) was compared between CRC and normal adjacent colonic tissue (*n* = 17), and found to be significantly increased in the carcinoma group (*P* = 0.0005, Figure 1A). Colon cancer follows the adenoma to carcinoma sequence, and most cancers arise from dysplastic adenomas. Therefore, we also examined *ACP1* expression levels in adenoma samples and again observed an increased mRNA expression in these samples (*n* = 32) compared to their normal adjacent colon tissue (*P* < 0.0001, Figure 1B).

### LMWPTP protein is overexpressed in primary colorectal cancer samples

Next, we examined whether the increased *ACP1* expression corresponds to increased protein levels of LMWPTP in CRC samples. Immunohistochemistry was performed on tissue sections of biopsies of low grade dysplasia (LGD; *n* = 9), high grade dysplasia (HGD; *n* = 7) adenocarcinoma (*n* = 12) and controls (*n* = 8) (Figure 1C). LMWPTP expression in intestinal epithelial cells (IEC) was limited to 9 ± 9% of cells in non-cancerous tissues. In contrast, expression of LMWPTP was significantly increased with subsequent levels of dysplasia (41 ± 33% and 80 ± 29% positive IEC in LGD and HGD, respectively), with up to 100% of LMWPTP-positive IECs in adenocarcinoma (Figure 1D). In addition to increasing numbers of positive cells, the intensity of the staining also increased in the untransformed-to-colorectal cancer sequence (0.44 ± 0.18, 0,72 ± 0.36, 1.50 ± 0.79 and 3.14 ± 0.90 in control, LGD, HGD, and CRC respectively, Figure 1E). Furthermore, LMWPTP overexpression is preserved in liver-metastasized CRC tumor cells, with 100% of IECs highly positive for this phosphatase (note that the normal liver tissue stains negative for LMWPTP) (*n* = 5).

**Figure 2:**
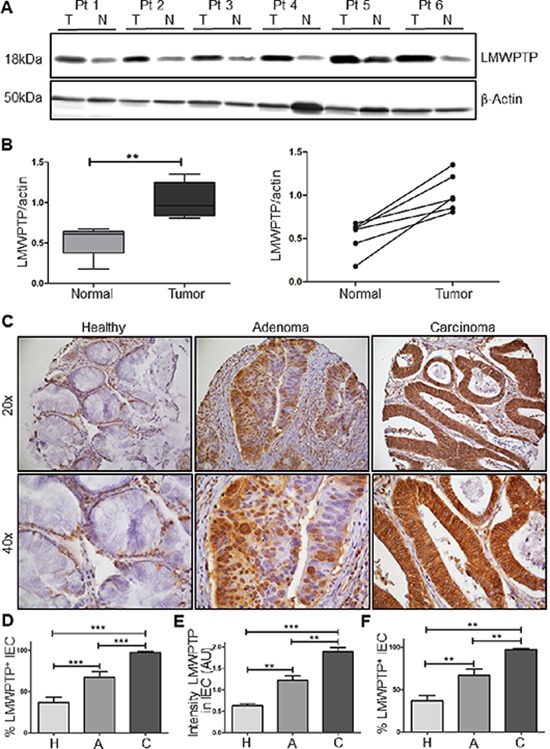
LMWPTP protein expression is increased in CRC as compared to paired normal adjacent tissue, and expression increases during the canonical progression sequence from normal tissue via adenoma to carcinoma **(A)** Freshly frozen paired tumor (T) and normal adjacent (N) colonic tissue samples of 6 patients were lysed in Laemmli buffer, and LMWPTP expression was determined using western blot analysis. β-Actin served as loading control. **(B)** Quantification of western blot results using Li-Cor Odyssey software presented as boxplot of mean, as well as comparisons of individual patient's samples (*P < 0.05; **P < 0.01). **(C)** IHC analysis of LMWPTP on a tissue micro array (TMA) of patients with colorectal cancer (*n* = 65), colorectal adenoma (*n* = 25), and healthy adjacent tissue (*n* = 62). Representative stainings (20x and 40X inset) of tissue cores from carcinoma, adenoma and healthy tissue are shown. **(D–E)** Staining was scored for percentage of LMWPTP positive intestinal epithelial cells as well as intensity of staining. Statistical analysis was performed using Mann-Whitney *t*-test. **(F)** Percentage of LMWPTP positive IEC were compared in patients from whom normal, adenoma and CRC tissue were all available (*n* = 15, Wilkinson paired *t*-test) (*P < 0.05; **P < 0.01; ***P < 0.001).

To validate these results using a different technique, we examined LMWPTP expression in 6 paired freshly frozen tumor and normal adjacent tissues by Western blotting, again demonstrating a significant increase in the total levels of this phosphatase in the tumor tissue (Figure 2A, 2B).

To confirm the increased LMWPTP protein expression in a larger sample group, the staining was subsequently performed on a tissue micro array (TMA) containing samples of 72 colorectal adenoma and/or carcinoma patients (Table 1; representative samples shown in Figure 2C). After excluding poor quality cores, 62 cores of CRC tissue, 25 cores of adenoma tissue and 65 cores of healthy adjacent tissue were available for analysis. Again, the cores were scored for percentage positive IECs and intensity of the staining. The mean percentage positive IEC was 27 ± 3% in normal adjacent tissues compared to 64 ± 4% in adenoma and 90 ± 3% in carcinoma (*P* < 0.001, Figure 2D). In addition, the intensity of the staining similarly increased from healthy tissue to adenoma and CRC (0.63 ± 0.05, 1.22 ± 0.10 and 1.90 ± 0.09, respectively, *P* < 0.001, Figure 2E). For 15 patients there was material available for all three stages. In these patients, a significant increase in LMWPTP expression from normal to adenoma, and adenoma to carcinoma tissue was observed (37 ± 6%, 67 ± 7% and 97 ± 1%, respectively, *P* < 0.001, Figure 2F), suggesting a role for LMWPTP in the oncogenic transformation of colonic epithelial cells. Due to the high expression of LMWPTP in all our carcinoma samples, we were unable to correlate clinical parameters such as Dukes' stage or patient survival to LMWPTP expression in cancer. However, when correlating patient survival to LMWPTP expression in their normal tissue, a higher LMWPTP expression in healthy tissue was significantly correlated to increased disease related mortality (Spearmans rho correlation *P* = 0.026, Supplementary Figure S1A). These data suggest either that there are infiltrating tumor cells present in what we denominate as normal adjacent tissue, or that an increased LMWPTP expression in normal cells is predictive or conductive to cellular transformation. Together, these results show that LMWPTP is overexpressed in a stepwise manner from normal tissue to carcinoma.

**Table 1:** Patients characteristics of tissue micro array

Parameter	Mean (SD) or N (%)
**Number of Patients**	72 (100%)
Healthy cores available	65 (90%)
Adenoma cores available	25 (35%)
Carcinoma available	62 (86%)
**Age at presentation**	
Mean	69.85 (11.8)
Median	70
Range	30–92
**Sex, N (%)**	
Male	37 (51.4%)
Female	35 (48.6%)
**Dukes' stage**	
A	1 (1%)
B	38 (47%)
C	23 (28%)
D	10 (12%)
**Status**	
Living	44 (61%)
Non-CRC-related death	13 (18%)
CRC-related death	15 (21%)

One of the mechanisms which may contribute to upregulation of LMWPTP expression levels, could be based on an altered methylation pattern of *ACP1*. Using an *in silico* analysis with the online database MENT (http://mgrc.kribb.re.kr:8080/MENT/) [15], we observed that *ACP1* is hypomethylated in colon cancer as compared to normal colonic tissue (*n* = 680; *P* < 0.0001), providing a possible explanation for the observed upregulation of gene expression (Supplementary Figure S1B).

### Effect of inhibition of LMWPTP on cell survival

**Figure 3:**
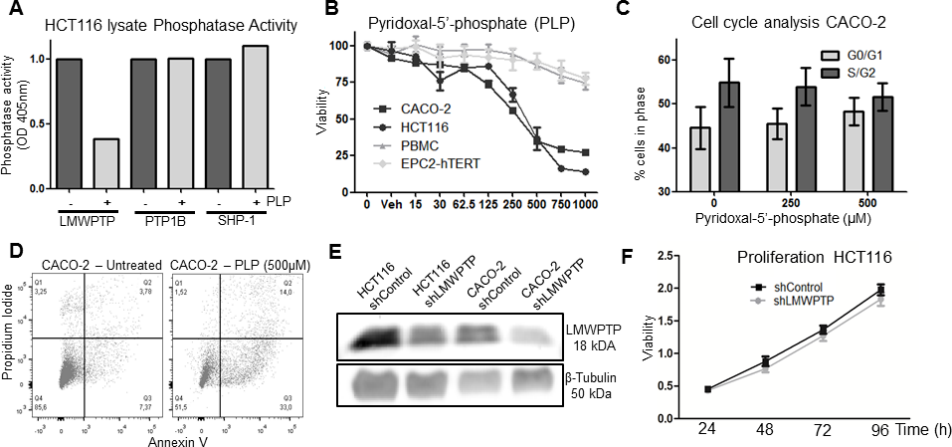
Effects of chemical inhibition and knockdown of LMWPTP on the oncogenic potential of colorectal cancer cells **(A)** Immunoprecipitated phosphatases (LMWPTP, PTP1B and SHP-1) from HCT116 lysates were incubated with the only known inhibitor of LMWPTP, PLP, resulting in reduction of LMWPTP phosphatase activity in LMWPTP precipitates, while enzymatic activity of the two other PNPP phosphatases remain unaffected upon PLP treatment. **(B)** Treatment of colorectal cancer cell lines (HCT116 and CACO-2) with PLP dose-dependently reduced viable cell numbers as determined by MTT assay, while non-transformed cell lines (EPC2-hTERT and PBMCs) are hardly effected. **(C)** Propidium-iodine staining of CACO-2 cells followed by FACS analysis shows that PLP treatment induces a G0/G1 cell cycle arrest. **(D)** PLP treatment of CRC cells results in apoptosis, as shown by FACS analysis with Annexin V/PI staining on CACO-2 cells treated either with 500 uM PLP or vehicle. **(E)** Stably transfected cell lines were created, by lentiviral transfection of HCT116 and CACO-2 cells with shRNA against LMWPTP. This resulted in cell lines harboring a knockdown of approximately 50% as compared to non-target transfected control cells. **(F)** LMWPTP knockdown does not affect overall cell proliferation as shown by MTT assay.

As our data in primary CRC indicates that an increased LMWPTP expression may contribute to tumor progression, we wondered whether inhibition of LMWPTP might reverse any of the oncogenic processes involved. The only LMWPTP inhibitor available to date is PLP, an active derivative of Vitamin B6, which has been shown to inhibit LMWPTP activity by interacting with the Asp129 site [16]. To confirm the effectiveness and selectivity of this compound, we precipitated LMWPTP and two other phosphatases with activity towards the substrate *PNPP* (SHP-1 and PTP1B) from CRC cells and demonstrated that the phosphatase activity of LMWPTP was indeed decreased in the presence of PLP, while the activity of SHP-1 and PTP1B were unaffected (Figure 3A and Supplementary Figure S2A). Next, we treated CRC cells (CACO-2 and HCT116), as well as the non-transformed human gastrointestinal epithelial cell line EPC2-hTERT and freshly isolated PBMCs, with this compound and assessed cell viability. As shown in Figure 3B, PLP dose-dependently reduced viable cell numbers of the CRC lines, while non-transformed cells are hardly affected by this treatment. PLP induced a G0/G1 cell cycle arrest in the cancer cells (Figure 3C and Supplementary Figure S2C). Furthermore, PLP treatment concomitantly caused apoptosis in CRC cells, as shown by an increased Annexin V staining (Figure 3D and Supplementary Figure S2B). Thus, these data suggest that chemical inhibition of LMWPTP may reduce CRC growth.

As PLP may have some off-target effects (LMWPTP is not the only molecular target to be inhibited by this compound), we decided to further investigate the effect of inhibition of LMWPTP by specifically reducing its expression. We employed shRNA against *ACP1* to stably knock down LMWPTP in HCT116 and CACO-2 cells, which reduced the expression of this phosphatase by 50% (Figure 3E). However, while chemical inhibition of LMWPTP affected cell viability and cell cycling, knockdown of LMWPTP did not (Figure 3F and Supplementary Figure S2D–S2F). This is perhaps not surprising, as knock down of LMWPTP was not complete, and the creation of stable cell lines would necessarily select for cells escaping cell death.

### LMWPTP induces drug resistance

**Figure 4:**
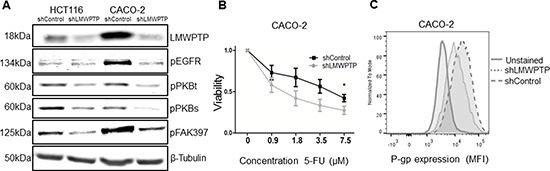
LMWPTP influences cell survival pathways and desensitizes colorectal cancer cells to chemotherapy **(A)** Western blot analysis of LMWPTP knockdown and control cells reveals reduced phosphorylation of Epidermal Growth Factor Receptor (EGFR), Protein Kinase B (PKBt and PKBs) and FAK in the knockdown cells. **(B)** CACO-2 LMWPTP knockdown and control cells were treated with increasing concentrations of 5-fluorouracil and cell viability was assessed by MTT assay after 96 h. CACO-2 knockdown cells were more susceptible to 5-fluorouracil as compared to control (*P < 0.05). **(C)** P-glycoprotein expression on CACO-2 cells was determined by FACS analysis, using anti-P-gp and anti-mouse-FITC antibodies. P-gp expression is reduced upon LMWPTP knockdown in CACO-2 cells, as shown by FACS mean fluorescence intensity (MFI).

Whilst not inducing cell death, knock down of LMWPTP in CRC lines allowed us to further investigate the role of LMWPTP in other oncogenic processes. We started by determining some of the molecular targets of LMWPTP. Figure 4A shows that knock down of LMWPTP resulted in the downregulation of several cancer-associated signaling pathways. Most noticeably, we observed a reduced phosphorylation of the epidermal growth factor receptor (EGFR) and diminished phosphorylation of protein kinase B (PKB) both on the threonine 308 and serine 473 sites in LMWPTP knockdown cells (Figure 4A). In addition to proliferation, these molecules are implicated in cell survival, and we therefor speculated that LMWPTP knockdown cells might be more susceptible to cytostatic agents. Indeed, treatment of CRC cells with 5-fluorouracil (5-FU), a commonly used chemotherapeutic, caused a dose dependent decrease in viable cell numbers, which was significantly more pronounced in LMWPTP knock down cells (Figure 4B and Supplementary Figure S3A). Thus, silencing of LMWPTP confers drug sensitivity of CRC cells, possibly through loss of EGFR and PKB activity. Other mechanisms used by tumor cells to escape drug effects include the expression and regulation of multidrug resistance efflux pumps. These include p-glycoprotein, also known as multidrug resistance protein 1 (MDR1), which transports several substrates across the extracellular membrane. Interestingly, P-gp was expressed on CACO-2 cells, and its expression was reduced upon silencing of LMWPTP (Figure 4C). In contrast, HCT116 cells did not express this particular efflux pump (and hence no decrease was observed in LMWPTP knock down cells), suggesting that different mechanisms may contribute to drug sensitivity in different CRC lines (Supplementary Figures S3B).

### LMWPTP targets migration signaling

**Figure 5:**
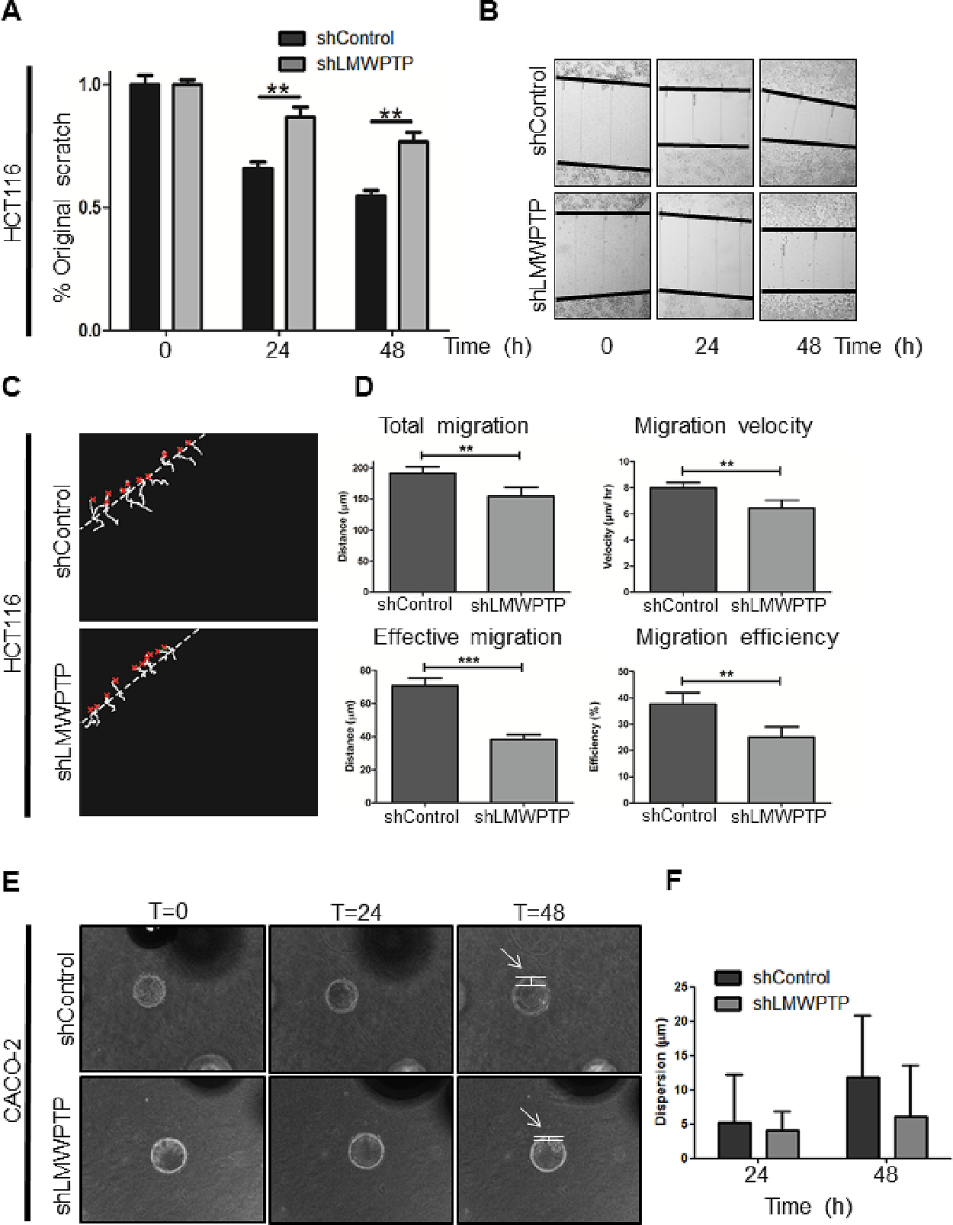
Modulation of LMWPTP results in impaired migration and invasion in colorectal cancer cells **(A, B)** HCT116 cell migration was measured by scratch assays, where simple scratch wounds were made using a pipet tip, and pictures are taken at 0 h, 24 h, and 48 h. Persistent area of clear plastic was measured and statistical analysis was performed using student's *T*-test. **(C, D)** Two-dimensional migration was analyzed using a ring-barrier system. HCT116 cell migration on gelatin was tracked during 24 h, with locations being captured using time-lapse microscopy every 12 min (x=start, line=cell track) (C). Quantification of migrated path indicates that the total migration and velocity were significantly reduced in LMWPTP knockdown cells. Effective migration and thereby efficiency are even further reduced. (D; *P < 0.05; **P < 0.01; ***P < 0.001). **(E, F)** Beads were coated with either CACO-2 LMWPTP knockdown or control cells for 24 hours, and embedded in a collagen gel matrix. Cells were allowed to invade the collagen matrix, and pictures were taking at 0 h, 24 h, and 48 h (examples in E). The cell dispersion from the bead (arrow) into the collagen matrix was measured, and a trend towards reduced invasion was observed in LMWPTP knockdown cells. Data represents at least four beads (F).

Colorectal cancer is a frequently fatal disease because of its high propensity to migrate and invade other tissues, preventing curative surgical treatment. Cellular migration is dependent on the tight regulation of assembly and disassembly of focal adhesion sites. This process is mediated the by the formation of a FAK–Src complex, and phosphorylation of FAK-associated substrates such as paxillin and p130^cas^, all known to be required for cell motility [17]. Our biochemical analysis of LMWPTP deficient cells revealed reduced FAK Tyr-397 phosphorylation in these cells, suggesting that LMWPTP may function in this pathway to promote FAK Tyr-397 phosphorylation and the formation of membrane extensions characteristic of migrating cells (Figure 2A). We therefore investigated the effects of LMWPTP downregulation in colorectal cancer cell lines on their ability to migrate. Confluent plates of CACO-2 and HCT116 cells were scratched using a pipet tip, and cell migration into the wound was assessed after 24 h and 48 h. LMWPTP knock down cells showed a significant delay in the ability to migrate into the empty space (Figure 5A–5B and Supplementary Figures S4A–S4B, N.B. that HCT116 is a slower migrating cell line). To verify the positive role of LMWPTP in cell migration, we used a second, different approach to investigate cellular movement, which does not rely on wounding the CRC monolayer. Using time-lapse microscopy of cell migration we again observed that CACO-2 and HCT116 LMWPTP knockdown cells are significantly impaired in their total migration, and thereby also the cell velocity. Strikingly, the effective migration, which is defined as the directional movement of the cells to the cell-free center, was even more reduced (Figure 5C–5D and Supplementary Figures S4C–S4D), Supplementary Movies). We subsequently went on to assess the role of LMWPTP on migration in a 3D-setting, representing the invasive capacity of these cells. Beads were coated with CACO-2 and HCT116 knockdown or control cells, and were settled in a collagen matrix. Cell dispersion from the bead into the surrounding collagen matrix was measured. Although not reaching statistical significance, we observed a trend towards reduced invasive capacity upon LMWPTP knock down for both cell lines (Figure 5E–5F and Supplementary Figure S4E–S4F). Together these data demonstrate that knocking down LMWPTP in colorectal cancer cells reduces their migratory capacity, and is especially important for directional cell migration.

### Different mechanisms for reduced migratory responses in CRC cells

**Figure 6:**
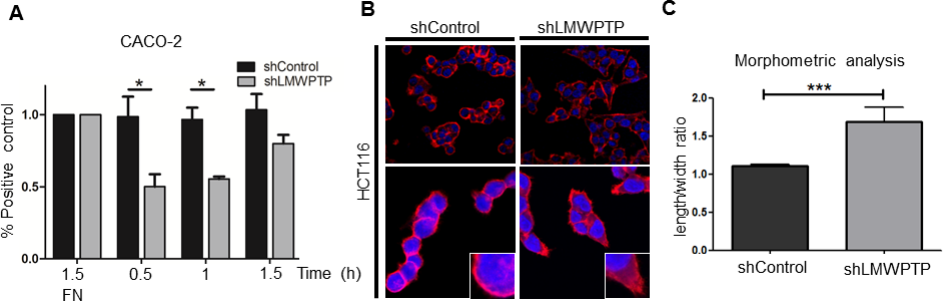
LMWPTP influences cell adhesion and cell morphology **(A)** CRC cell adhesion was determined by MTT assay of adherent cells after indicated time points, with fibronectin (FN) coating serving as control. CACO-2 LMWPTP knockdown cells adhere less than control cells (*P < 0.05; **P < 0.01). **(B, C)** Confocal microscopy of Phalloidin-rhodamine stained cells was employed to examine cell morphology. Immunofluorescence reveals a more elongated morphology in HCT116 LMWPTP knockdown cells as compared to control (B). To quantify the changes in morphology, the ratio between the length and width of the cells was calculated, with a higher ratio indicating a more elongated shape. Non-target control cells have a significantly lower ratio compared to LMWPTP knockdown cells. (1.1 ± 0.02 vs 1.7 ± 0.18, *n* = 26; *P* < 0.0001) (C).

As both CACO-2 and HCT116 cell lines demonstrated reduced FAK activity and subsequent migration upon LMWPTP knock down, we next set out to further examine the underlying mechanism of this reduced migration. First we investigated the adhesive capacity of these cell lines, adhesion being indispensable for proper migration, and FAK being a major regulator of this process. As shown in Figure 6A, full adherence of CACO-2 non-target cells to the glass surface was reached after 30 minutes and 1 h, while only 50% of the knockdown cells adhered to the bottom of the wells within these time points (*P* > 0.05). In contrast, HCT116 cells were much slower to adhere, and no differences could be observed between LMWPTP knock down and control cells (Figure S5A).

Next, we employed confocal microscopy to investigate the actin distribution as well as cellular morphology of the cells. We found alterations in the cytoskeletal F-actin composition in HCT116 cells downregulated for LMWPTP. Loss of LMWPTP resulted in a more stretched appearance of cells, suggestive of a less migratory phenotype (Figure 6B). This is also reflected by our morphometric analysis (Figure 6C), which reveals a significantly higher length/width ratio in the LMWPTP knockdown cells (1.1 ± 0.02 in control cells and 1.7 ± 0.18 knockdown cells; *P* < 0.0001). In contrast, CACO-2 cells, which have a cuboidal appearance, did not show the stretching in the knockdown cells observed in the HCT116 cell line (Supplementary Figure S5B). These results indicate that while the net effect of LMWPTP on migration is similar in CACO-2 and HCT116 cell lines, the underlying mechanisms differ, emphasizing the heterogeneity of CRC.

## DISCUSSION

In this study we identify LMWPTP overexpression as a mediator for increased chemoresistance and migration in colorectal cancer. We show for the first time, that LMWPTP protein expression is drastically increased in primary colorectal cancer samples as compared to normal adjacent tissue. LMWPTP expression appears to follow a step-wise increase from healthy tissue, to dysplastic adenoma, and to carcinoma. Deregulated transcription and translation is a common phenomenon of cancer cells, and many signaling pathways can concomitantly be affected in malignant cells. However, analysis of other phosphatases in CRC sections does not indicate a general upregulation of PTPs in this tumor type (unpublished results), indicating a specific oncogenic role for LMWPTP in the transformation of colon epithelial cells. While the cause of the specific upregulation of *ACP1* mRNA has yet to be identified, interestingly, an initial database analysis (MENT methylation and expression database) suggests that this gene is hypomethylated in adenoma and cancer samples.

Thus far, while several phosphatases have been shown to act as tumor suppressors, few phosphatases have been implicated as oncogenes in colorectal cancer. Phosphatase of regenerating liver-3 (PRL-3), also known as PTP4A3, was shown to be upregulated in up to 100% of CRC liver metastasis cases, and is overexpressed in primary tumors [18]. Furthermore, PRL-3 can promote migration and invasion by enhancing MMP2 activity [19]. Similarly, the phosphatase SAP-1 has been shown to be overexpressed in colorectal tumors, and especially promotes the tumorigenic potential of intestinal epithelial cells [20, 21]. Likewise, PTPα is capable of activating Src protein kinase activity in colorectal cancer, further supporting the idea that phosphatases not only act as inhibitors of oncogenic kinases, but can function as tumor promoters as well [22]. To this relatively short list, we may now add LMWPTP. Although these other phosphatases may also be implicated in the colorectal cancer process, convincing demonstration of their dysregulation in a large cohort of well-characterized colon cancer patients as well as detailed mechanistic insight into their exact role in the cancer process is largely lacking. In contrast, LMWPTP appears a bona-fide mediator of the CRC invasiveness and chemoresistance.

After our initial finding that LMWPTP is significantly overexpressed in colorectal cancer, we evaluated the effect of LMWPTP *in vitro*. Up to now, the most potent compound known to inhibit LMWPTP activity is the active form of vitamin B6, PLP. Interestingly, earlier reports have shown that both vitamin B6 and PLP serum levels are inversely correlated with colorectal cancer risk [23]. Our data suggest that inhibition of LMWPTP through Vitamin B6 may provide one possible explanation for these findings. Indeed, in pituitary cells PLP has been shown to induce cell cycle arrest [24], which corresponds well with the cell cycle block and apoptosis observed in our study, and suggests that LMWPTP inhibition may be a valuable avenue for treatment of CRC. Earlier reports have suggested that LMWPTP could modulate chemoresistance of cancer cells [25]. The current treatment protocol for advanced CRC contains chemotherapy, such as 5-fluorouracil. Typically, the first course of chemotherapy is highly beneficial, but tumor cells tend to make use of several mechanisms to escape the therapy and become resistant. We now show that LMWPTP could be involved in these mechanisms in CRC. In addition to an increased sensitivity to chemotherapy, LMWPTP knock down cells showed a great reduction in phosphorylated EGFR and PKB, making it tempting to speculate that decreased activity of these survival signals contributes to drug-sensitivity of these cells. In addition, multidrug resistance pumps, such as P-gp are often upregulated on cancer cells and can contribute to decreased drug-sensitivity of tumors [26, 27]. Our study suggests that LMWPTP can act on these transporters as well, thereby contributing to the chemoresistance of CRC cells.

In this study, we observed clear differences between HCT116 and CACO-2 cells. While LMWPTP knock down conferred drug sensitivity in both cell lines, no modulation of P-gp was observed in HCT116 cells, suggesting different underlying mechanisms. In addition, while migratory and invasive behavior was impaired in both LMWPTP knock down cell lines, the molecular pathways contributing to migratory defects appeared to differ. Both cell lines demonstrated reduced phosphorylation of the focal adhesion kinase FAK. This kinase, by forming a complex with p120RasGAP and p190RhoGAP (p190A), leads to phosphorylation of p190A, resulting in polarity cues and increased directional movement [28]. Impaired formation of the Ras-Rho complex as a result of LMWPTP downmodulation may thus contribute to the inefficient cell movement and polarity observed in our migration assays. FAK is a major regulator in the adhesion to matrix, which is also essential for proper migration. However, while we observed adhesion defects in LMWPTP knock down CACO-2 cells, the same was not observed in HCT116 cells. This second LMWPTP knock down cell line selectively demonstrated a changed morphology and F-actin rearrangement, characteristic for sessile behavior of cells. These latter findings are reminiscent of (colorectal) cancer cells in which a more rounded morphology as a result of overexpression of RhoA correlated to increased ability to migrate *in vitro* and metastasize *in vivo* [29]. Thus, while inhibition of LMWPTP in both of these colorectal cancer lines appears beneficial in terms of reducing cell growth, drug resistance and metastatic potential, the mechanisms through which this is achieved may rely on genetic identity of the tumor cells. These data highlight the need for personalized medicine in cancer treatment, as different genetic backgrounds may affect the usefulness of treatment regimens as well as the molecular mechanisms behind them. This was recently very clearly demonstrated by the report of a selective benefit of mTOR inhibitors only in patients carrying PTEN-deficient tumors [30].

In summary, we show that low molecular weight protein tyrosine phosphatase is overexpressed in primary human colorectal cancers at both mRNA and protein level and that this phosphatase can function as an oncogene, by enhancing the migration, adhesion and chemoresistance in colorectal cancer cells. Together, this indicates that LMWPTP expression is a determining factor in the malignant potential of colorectal cancer, and suggests that this phosphatase provides a target in the fight against this devastating disease.

## MATERIALS AND METHODS

### Gene expression profiles

Expression profiles from publicly available NCBI GEO datasets were browsed to find comparisons of CRC or colorectal adenoma samples to their adjacent normal tissue. Information on *ACP1* expression was available in 2 arrays. Dataset Record GDS4382 (transcript 215227_x_at), based on the Affymetrix Human Genome U133 Plus 2.0 Array, was used to compare 17 paired CRC and adjacent normal tissue samples [31]. The same platform was used in dataset record GDS2947 (transcript 215227_x_at), used to compare 32 paired colorectal adenoma and adjacent normal tissue samples [32]. *P*-values were calculated per probe using Student's *t*-tests.

### Patient selection

At the Erasmus MC Formalin fixed paraffin embedded (FFPE) colorectal tissue specimens were collected from the department of pathology for 9 low grade dysplasia (LGD) patients, 5 high grade dysplasia (HGD) patients, 7 adenocarcinoma (CRC) patients and 5 patients with CRC-related liver metastasis. Patients with active and inactive ulcerative colitis (*n* = 8) served as controls.

In addition, a tissue micro array was constructed at the Leiden University Medical Centre (LUMC), containing material from 72 patients with colorectal cancer. Representative cores of healthy adjacent tissue were available for 65 patients, 25 patients had available adenoma cores, and 62 patients had representative carcinoma cores.

### Immunohistochemistry

The FFPE tissue sections and TMA were immunohistochemically stained for LMWPTP (Acp1 antibody, sc-100343, Santa Cruz Biotechnologies, Dallas, Tx) as described [33]. Briefly, 5 μm sections were deparaffinized in xylene and rehydrated through graded alcohols. Antigen-retrieval was performed by boiling the slides in citrate buffer pH 6.0 for 15 minutes. Endogenous peroxidases were blocked by immersing the slides for 10 minutes in 3% H_2_O_2_ in phosphate buffered saline (PBS). Next, slides were blocked by incubation in PBS containing 10% goat serum in for 1 h at RT. Primary antibody was added 1:100 in blocking buffer and incubated overnight at 4°C. Envision goat anti-mouse-horseradish peroxidase (Dako, Heverlee, Belgium) was used as secondary antibody. The slides were scored for the percentage of positive epithelial cells as well as intensity of the staining on a 4 scale scoring system. *P*-values were calculated using Student's *t*-tests and Wilcoxon signed-rank test for the paired samples.

### Cell lines

HCT116 and CACO-2 colorectal cancer cells were purchased from ATCC (Manassas, USA) and cultured in Dulbecco's Modified Eagles Medium (DMEM, Lonza, Basel, Switzerland), supplemented with 10% fetal bovine serum (Sigma-Aldrich, St. Louis, USA). EPC2-hTERT cells are cultured in Keratinocyte-SFM medium (Life technologies, Bleiswijk, NL), supplemented with Epidermal Growth Factor (EGF) and Bovine Pituitary Extract (BPE). All cell cultures were supplemented with 100 U/ml penicillin, 100 mg/ml streptomycin (Life technologies, Bleiswijk, NL), and propagated at 37°C in a 5% CO2 humidified atmosphere. Cell lines were routinely tested for Mycoplasm infection using MycoAlert (Lonza, Basel, Switserland).

### Cell culture and transfections

Using a lentiviral system, stably transfected LMWPTP knockdown cells were generated. In brief, HEK293T cells were transfected with LMWPTP or non-target control shRNA (Sigma-Aldrich, St. Louis, USA) and viral plasmid, generating virus containing medium. CRC cells were incubated with the conditioned medium for 48 hours after which transfected cells were selected using puromycin (2 μg/ml, Sigma-Aldrich, St. Louis, USA).

### Western blotting

Subconfluent cells were lysed on ice in 2x concentrated Laemmli buffer (100 mM Tris–HCl (pH 6.8), 200 mM dithiothreitol, 4% SDS, 0.1% bromophenol blue and 20% glycerol) and samples were boiled for 10 min. Cell extracts were resolved by SDS–PAGE and transferred to polyvinylidene difluoride membranes (Merck chemicals BV, Amsterdam, the Netherlands). Membranes were blocked in 50% odyssey blocking buffer (LI-COR Biosciences, Lincoln, NE) in PBS/0.05% Tween-20 and incubated overnight at 4°C with primary antibody. After washing in PBS-T, membranes were incubated with IRDye® *antibodies* (LI-COR Biosciences, Lincoln, NE) for 1 h. Detection was performed using Odyssey reader and analyzed using manufacturers software. For antibodies used see Supplementary Table 1.

### Immunoprecipitation and phosphatase assay

To quantify the phosphatase activity, cells were lysed with 200 μl of Lysis Buffer (20 mM HEPES, pH7.7 with 2.5 mM MgCl2, 0.1 mM EDTA, 1 mM PMSF, 1 mM DTT, 10 μg/mL aprotinin and 10 μg/mL leupeptin) on ice for 2 h. After clarifying by centrifugation, the cell extracts were incubated overnight at 4°C under rotation with antibodies against LMWPTP (Acp1 antibody, sc-100343, Santa Cruz Biotechnologies, Dallas, Tx) PTP1B (PTP1B antibody, sc-14021, Santa Cruz Biotechnologies, Dallas, Tx) or SHP-1 (SH-PTP1 antibody, sc-7289). A/G-Sepharose beads were added to cell homogenates and incubated for 2 h at 4°C. Cell extracts were washed 3 times with lysis buffer and 2 times with acetate buffer (100 mM pH5.5). The precipitate was resuspended in acetate buffer containing *PNPP* as substrate. 45 min after incubation at 37°C, equal volume of 1N HCl was added, and phosphatase activity was measured using a spectrophotometer at 405 nm.

### Cell viability assay

Using a colorimetric MTT (3-(4,5-Dimethylthiazol-2-yl)-2,5-diphenyltetrazolium bromide assay, proliferation was measured. In brief, 10.000 cells were seeded in 96 wells plate, after 24, 48, 72 or 96 hours 5 mM MTT was added, and incubated for 2 hours. Next, cells were resuspended in 100 μl of Dimethyl sulfoxide and wavelength was measured using a spectrophotometer at 490 and 595 nm. Each assay was performed at least three times in duplicate.

### Cell cycle analysis

Cell cycle analysis was performed by staining the cells for 1 h in sodiumcitrate-dihydrate (1 g/L) solution, containing 20 μg/mL propidiumiodide, 0.1% triton-X100 and 100 μg/mL ribonuclease A. The cell cycle distributions were analyzed using Modfit LT software. Each assay was performed twice.

### P-Glycoprotein expression levels

P-gp expression levels were measured using standard flow cytometric analysis. Cells were incubated with primary anti-mouse P-gp antibody (Imunnotech, Marseille, France). After washing, cells were incubated with anti-mouse FITC labelled antibody, and analyzed by flow cytometry. Data is shown as mean fluorescence intensity (MFI).

### Adhesion assay

50.000 cells were loaded into 96 wells plates and allowed to adhere for different time points to the plate surface either coated with fibronectin, or without coating. After 30 min, 1 h and 1.5 hours non-adherent cells were washed away. After 1.5 hours, MTT was added to the plate in order to quantify the amount of adhered cells. Cells adhering to fibronectin coating for 1.5 hours served as control. Assays were performed twice, with 8 duplicates averaged in each assay.

### Scratch migration assay

Cells in 6-well plates were grown to semi confluence. Using a yellow Gilson pipette tip simple scratch wounds were made. After washing the cells, the persisting areas of clear plastic were measured at 0, 24 and 48 hours using Axiovision 3.0 software (Carl Zeiss Vision GmbH) and the reduction in scratch wound area from time 0 was calculated. Each assay was performed twice, in duplicate.

### Cell migration assay “ring barrier system”

Cell migration assays were performed using the ring-barrier migration assay previously described [34]. Briefly, sterile coverslips placed in an Attofluor incubation chamber were coated with gelatin (1 mg/ml) and incubated for 1 h at 37°C, prior to cell seeding. A removable circular sterile migration barrier was inserted into the chamber, which prevents cell growth in the center of the coverslip. 4×10^5^ HCT116 and 2,5×10^5^ CACO-2 knockdown and control cells were seeded around this barrier and the rings were incubated at 37°C for 24 h, thereby generating a confluent monolayer in the periphery and a cell-free area in the center of the coverslip. After removing the migration barrier, time-lapse imaging was conducted at 37°C under humidified 5% CO_2_ air flow for 24 h on an Axiovert 100M inverted microscopes, equipped with an AxioCam MRC digital cameras, using a 10X/0.30 Plan-Neofluar objective (Carl Zeiss B.V., Sliedrecht, Netherlands). Time-lapse movies (images taken every 12 min) were used to quantify cell migration using AxioVision 4.5 software. For each movie, 10 cells at the migration front were randomly selected and tracked for the analysis. The net track movement of cells in 24 h was termed ‘total migration’, while the directional movement of cells to the cell-free center of the coverslip was termed ‘effective migration’. Migration efficiency was determined as the percentage of directional movement over the total track distance. For each cell line, at least three independent migration assays were performed.

### 3D-migration using cell dispersion assay

Cytodex-3 microcarrier beads (Sigma–Aldrich) were mixed with 5×10^5^ CACO-2 and HCT116 knockdown and control cell suspensions, considering a density of 40 cells per bead, and incubated at 37°C for 6 h with gentle mixing. The bead suspension was transferred to a 25 cm^2^ tissue culture flask and incubated for 48 h to ensure complete coating of beads and to remove unattached cells. Coated beads were embedded in 1.6 mg/ml collagen gel (collagen: modified Eagle's medium:7.5% w/v NaHCO_3_ in the ratio 8:1:1) in a 24-well plate such that each well had approximately 150 beads. Plates were incubated at 37°C for 2 h for the beads to settle in the gel and the polymerized gels were covered with 500 μl DMEM, 10% FBS, 1% p/s. Cell dispersion was measured as the maximum migrated distance from the surface of the bead into the collagen gel. All measurements were performed using AxioVision 4.5 software and assays were performed three times in duplicate. Two-way analysis of variance was performed to calculate *P*-values.

### Immunofluorescence

Subconfluent cells, cultured on glass coverslips, were fixed 15 min in 4% paraformaldehyde in PBS and permeabilized in 0.1% Triton-X100 in PBS (PBS-T). Actin filaments were stained with 10 μg/mL phalloidin-TRITC in PBS-T. Cell nuclei were stained with 200 ng/mL DAPI (4′, 6′-diamidino-2-phenylindole) in PBS-T for 30 min and coverslips were mounted using Vectashield mounting medium. Immunofluorescent images were taken using Zeiss LSM510Meta confocal microscope with x40 OilFLUAR lens. Morphometric analysis was performed by measuring the length/width ratio of 26 randomly selected cells.

## SUPPLEMENTARY FIGURES AND TABLE


