# Supplementary material for: Low molecular weight protein tyrosine phosphatase (LMWPTP) upregulation mediates malignant potential in colorectal cancer
Source: Oncotarget. 2015 Mar 14;6(10):8300–12. doi: 10.18632/oncotarget.3224 (PMC4480753; doi:10.18632/oncotarget.3224)
Supplement: Supplementary file 1 [file oncotarget-06-8300-s001.pdf]

SUPPLEMENTARY FIGURES AND TABLE

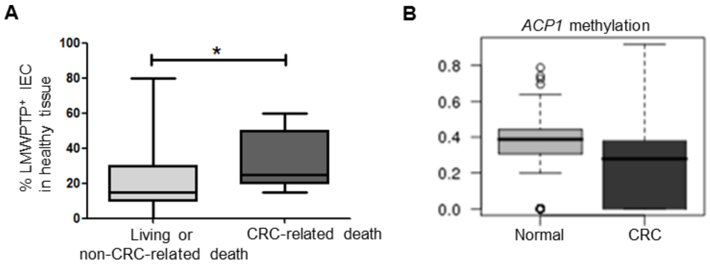

Supplementary Figure 1: LMWPTP upregulation is related to *ACP1* hypo-methylation and corresponds to patient survival in normal adjacent colonic tissue. **(A)** Analysis of percentage LMWPTP positive IECs in normal adjacent tissue cores, related to patient outcome. Patient group is divided in patients still alive or suffering a non-CRC-related death, and patients deceased due to colorectal cancer. **(B)** *In silico* analysis of *ACP1* methylation in colorectal cancer and normal colonic tissue, using MENT database. *ACP1* is significantly hypo-methylated in colorectal cancer as compared to normal tissue (−0.24032; $P > 0.00001$).

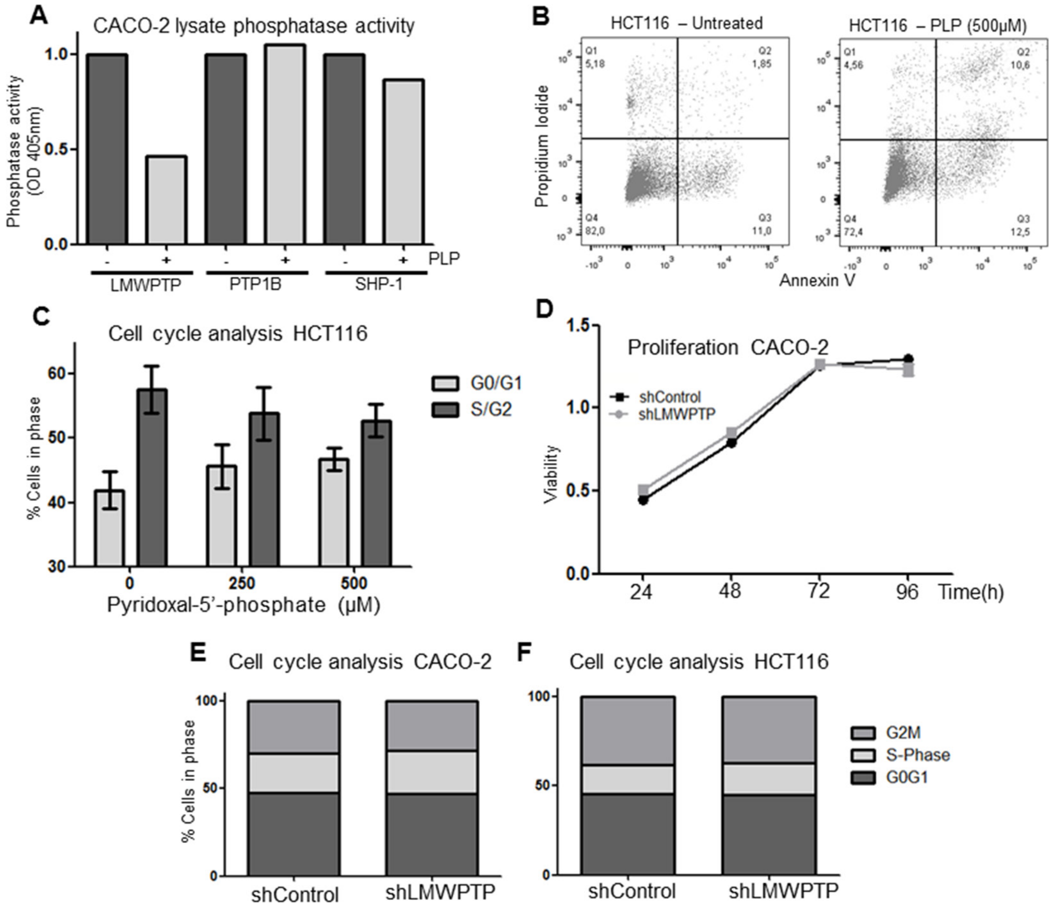

Supplementary Figure 2: Effects of chemical inhibition and knockdown of LMWPTP on colorectal cancer cells. **(A)** Immunprecipitated phosphatases (LMWPTP, PTP1B and SHP-1) from CACO-2 lysates were incubated with the only known inhibitor of LMWPTP, PLP, resulting in reduction of LMWPTP phosphatase activity, while the two other PNPP phosphatases remain unaffected upon PLP treatment. **(B)** Treatment of HCT116 cells with 500 uM of PLP induces apoptosis as determined by Annexin V/Pi FACS analysis. **(C)** Treatment of HCT116 cells with PLP induces cell cycle arrest as determined by ploidity through FACS analysis of propidium iodide staining. **(D)** MTT analysis shows that stable knockdown of LMWPTP in CACO-2 cells does not affect viable cell numbers in culture. **(E, F)** Analysis of cell cycle by propidium iodide staining shows no differences between LMWPTP knockdown and control cells for CACO-2 **(E)** and HCT116 **(F)** CRC cell lines.

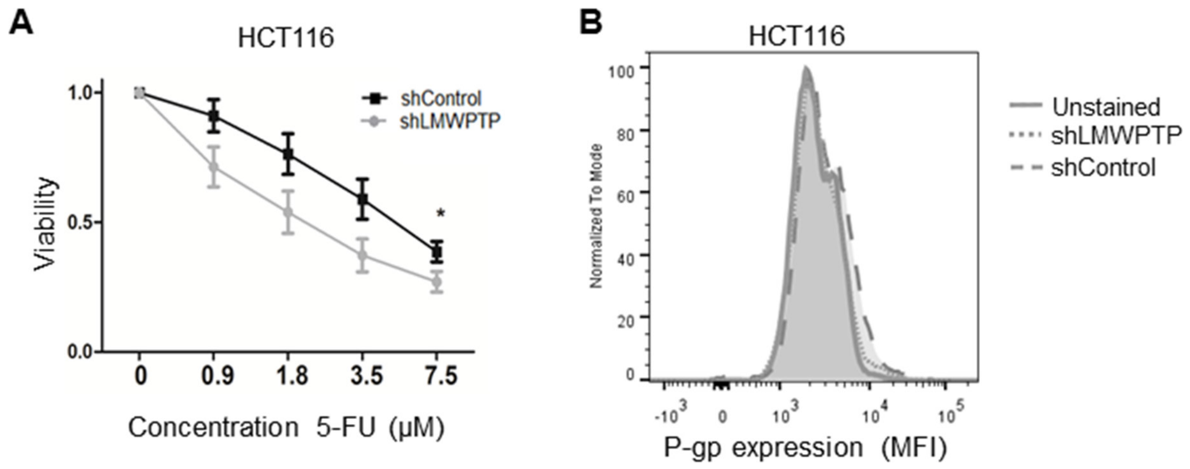

Supplementary Figure 3: LMWPTP knockdown sensitizes colorectal cancer cells to chemotherapy. (A) HCT116 knockdown and control cells were treated with increasing concentrations of 5-fluorouracil (5-FU) for 96 hours. Knockdown cells are more susceptible to 5-fluorouracil treatment as compared to non-target control cells. **(B)** In contrast to CACO-2 cells, HCT116 cells show little P-glycoprotein expression, as determined by FACS analysis.

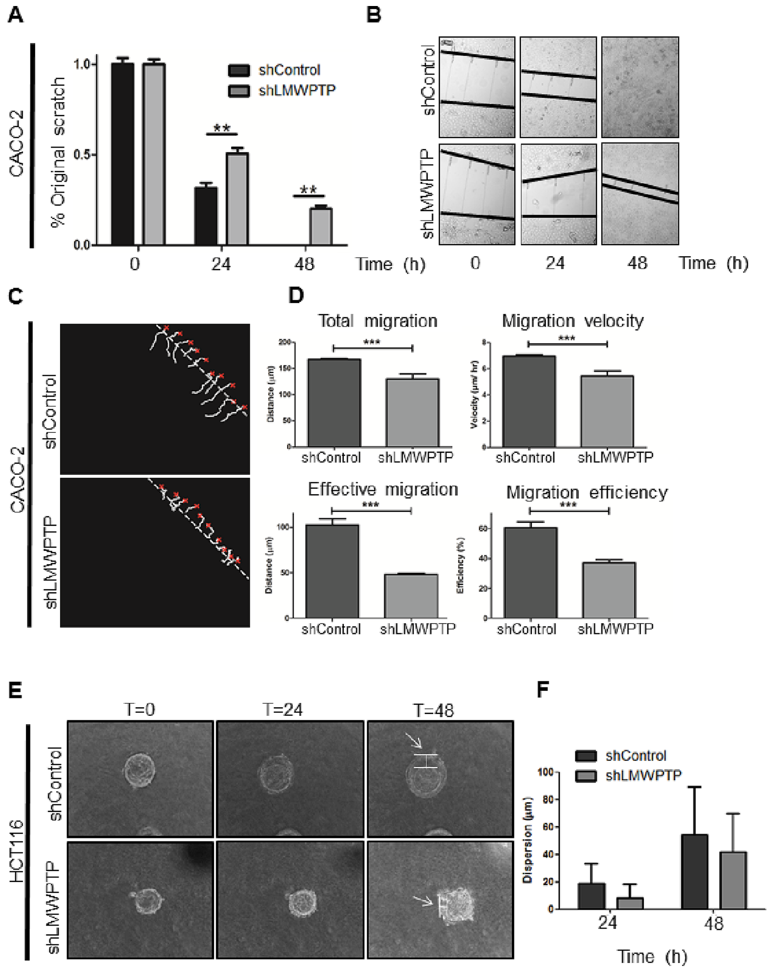

Supplementary Figure 4: Downmodulation of LMWPTP results in reduced migration and invasion in colorectal cancer cells. (A, B) CACO-2 cell migration was measured by scratch assays, where simple scratch wounds were made using a pipet tip, and pictures are taken at 0 h, 24 h, and 48 h. Persistent area of clear plastic was measured and statistical analysis was performed using student's *T*-test. **(C, D)**. Two-dimensional migration was analyzed using a ring-barrier system. CACO-2 cell migration on gelatin was tracked during 24 h, with locations being captured using time-lapse microscopy every 12min (x=start, line=cell track)(C). Quantification of migrated path indicates that the total migration and velocity were significantly reduced in LMWPTP knockdown cells. Effective migration and thereby efficiency are even further reduced. (D; $*P < 0.05$; $**P < 0.01$; $***P < 0.001$). **(E, F)** Beads were coated with either HCT116 LMWPTP knockdown or control cells for 24 hours, and embedded in a collagen gel matrix. Cells were allowed to invade the collagen matrix, and pictures were taking at 0 h, 24 h, and 48 h (examples in E). The cell dispersion from the bead into the collagen matrix (arrow) was measured, and a trend towards reduced invasion was observed in LMWPTP knockdown cells. Data represents at least four beads **(F)**.

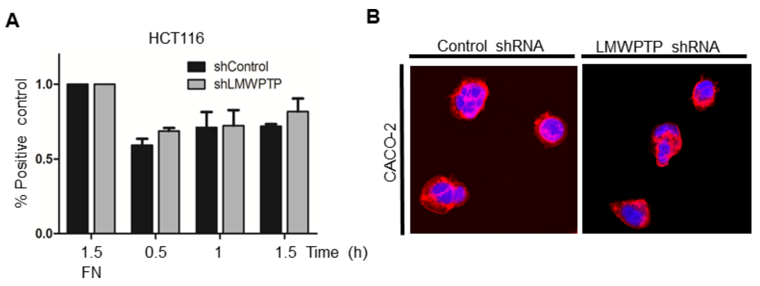

Supplementary Figure 5: LMWPTP influences cell adhesion and cell morphology. (A) CRC cell adhesion was determined by MTT assay of adherent cells at indicated timepoints, with fibronectin (FN) coating serving as control. HCT116, a slow migrating cell line, does not show reduced adhesion upon LMWPTP knockdown. **(B)** Confocal microscopy of Phalloidin-rhodamine stained CACO-2 cells was employed to examine cell morphology. The more stretched appearance upon knockdown of LMWPTP in HCT116 cells was not observed in CACO-2 cells, which have a more cuboid appearance, form clumps when cell numbers are low and grow out as complete sheets as cell numbers increase.

Supplementary Table 1: Summary of all primary and secondary antibodies used

Antibody	Dilution	Company	Catalog number
**Primary antibodies**			
Mouse-anti-ACP1α/β	1:500	Santa Cruz	sc-100343
Rabbit-anti-Tubulin	1:10000	Abcam	Ab-6046
Mouse-anti-Actin	1:2500	Santa Cruz	Sc-47778
Rabbit-anti-phospho-AKT (T308)	1:1000	Cell signaling	#2965
Rabbit-anti-phospho-AKT (S473)	1:1000	Cell signaling	#9271
Rabbit-anti-phospho-EGFR (Y1068)	1:1000	Cell signaling	#3777
Rabbit-anti-phospho-FAK (Y397)	1:1000	Invitrogen	44-625G
P-Glycoprotein	1:20	Immunotech	1864
Phalloidin-Rhodamin	1:200	Invitrogen	R415
DAPI (4’, 6’-diamidino-2-phenylindole)	1:1000	Sigma	D9542
**Secondary antibodies**			
**Antibody**	**Dilution**	**Company**	**Catalog number**
HRP-conjugated goat-anti-mouse IgG (EnVision™)	n/a	DAKO	K4007
HRP-conjugated goat-anti-Rabbit IgG (EnVision™)	n/a	DAKO	K4011
Goat-anti-rabbit IgG IRDye 800	1:10000	Westburg	926–32211
Goat-anti-rabbit IgG IRDye 680	1:10000	Westburg	926–68072
Goat-anti-mouse IgG DyLight 488	1:50	Biolegend	405310

SUPPLEMENTARY VIDEOS

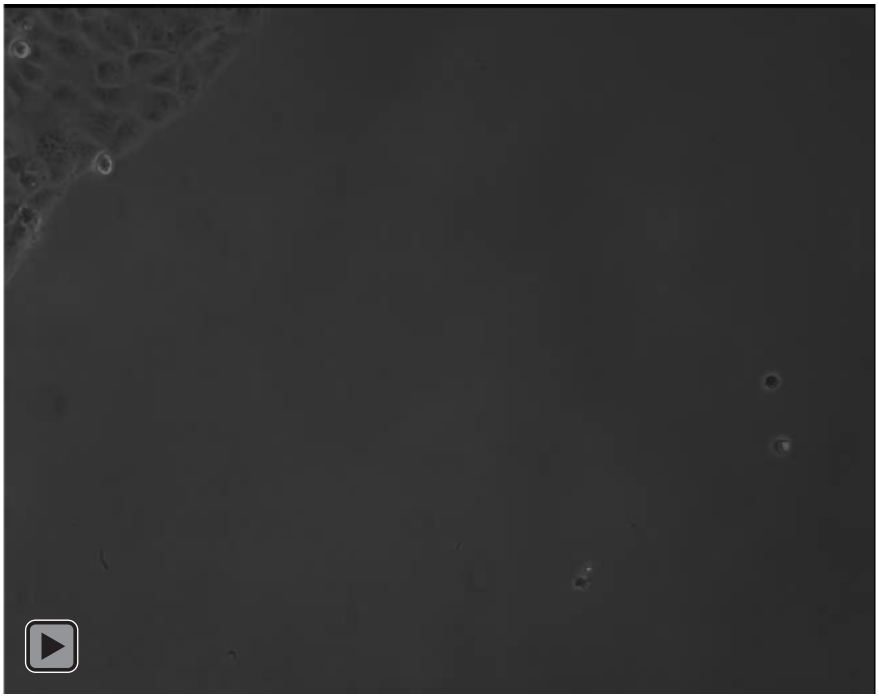

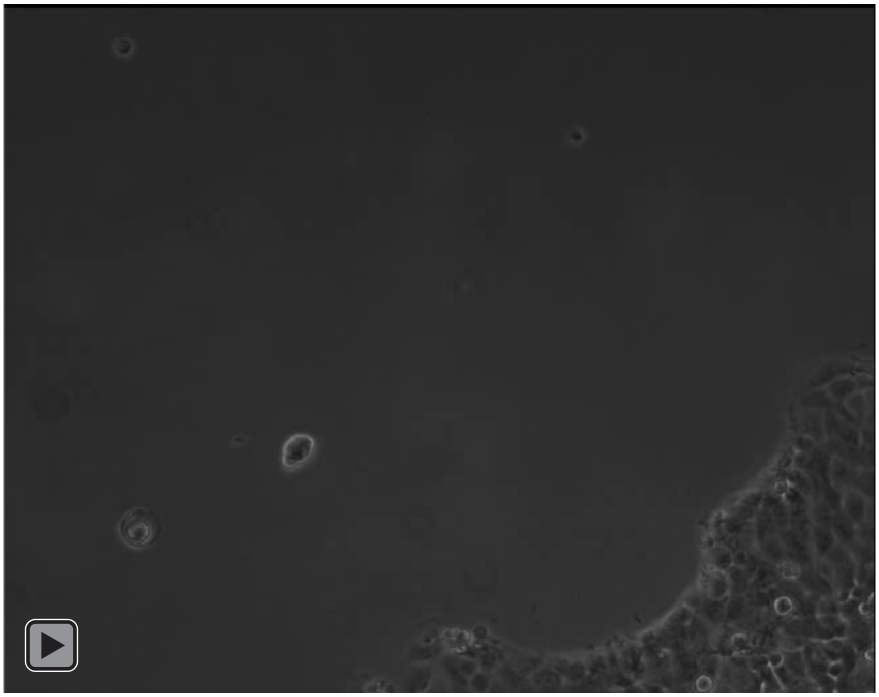

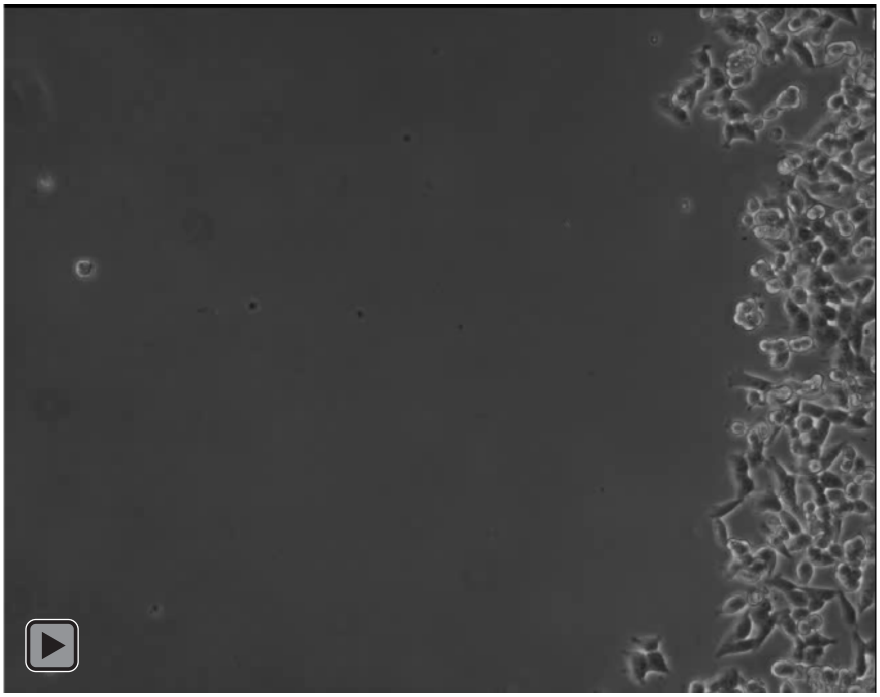

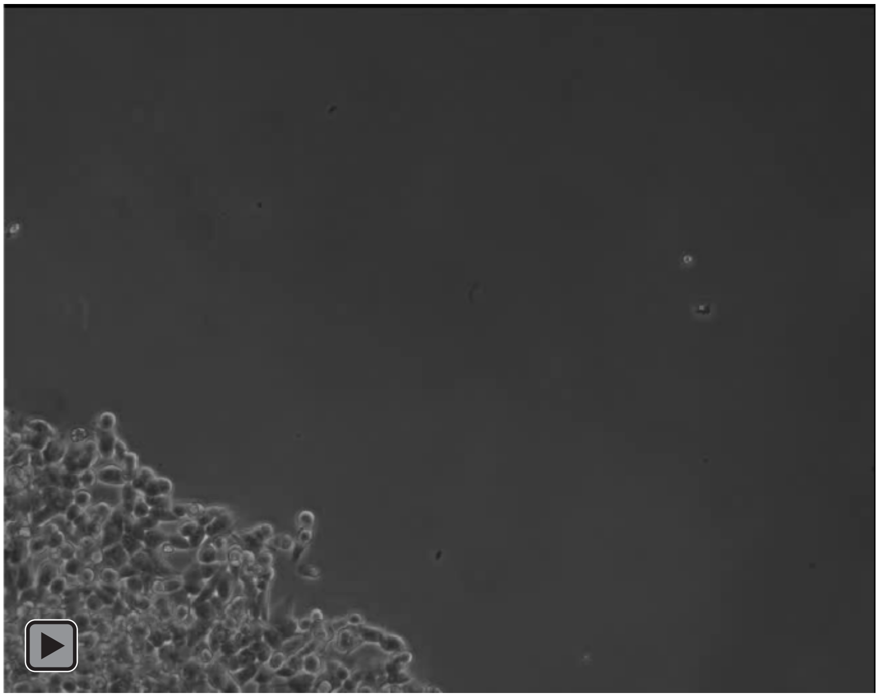

Supplementary Movies S1–S4: Downmodulation of LMWPTP results in reduced migration. Time-lapse movies (images taken every 12 min) of cell migration in CACO-2 (S1–S2) and HCT116 (S3–S4) control and knockdown cells. LMWPTP knockdown cells (S1 and S3) migrate significantly less as compared to control cells (S2 and S4). The impairment of the directional movement is even more pronounced.
